# Enhancing the nutritional profile of purple sweet potato flour through fermentation with *Lactiplantibacillus plantarum* InaCC B157: A functional food perspective

**DOI:** 10.14202/vetworld.2025.1870-1880

**Published:** 2025-07-11

**Authors:** Tiurma Pasaribu, Yati Sudaryati Soeka, Novik Nurhidayat, Suciatmih Suciatmih, Titin Yulinery, Evi Triana, Tri Ratna Sulistiyani, Ninik Setyowati, Desty Dwi Sulistyowati, Dwi Ningsih Susilowati

**Affiliations:** 1 Research Center for Animal Husbandry, National Research and Innovation Agency, Jl. Raya Jakarta-Bogor No.32, Pakansari, Kec. Cibinong, Kabupaten Bogor, West Java 16915, Indonesia; 2 Research Center for Applied Microbiology, National Research and Innovation Agency, Jl. Raya Jakarta-Bogor No.32, Pakansari, Kec. Cibinong, Kabupaten Bogor, West Java 16915, Indonesia; 3 Research Center for Biosystematics and Evolution, National Research and Innovation Agency, Jl. Raya Jakarta-Bogor No.32, Pakansari, Kec. Cibinong, Kabupaten Bogor, West Java 16915, Indonesia; 4 Research Center for Applied Botany, National Research and Innovation Agency, Jl. Raya Jakarta-Bogor No.32, Pakansari, Kec. Cibinong, Kabupaten Bogor, West Java 16915, Indonesia; 5 Research Center for Horticulture, National Research and Innovation Agency, Jl. Raya Jakarta-Bogor No.32, Pakansari, Kec. Cibinong, Kabupaten Bogor, West Java 16915, Indonesia

**Keywords:** amino acids, fatty acids, fermentation, functional food, *Lactiplantibacillus plantarum*, purple sweet potato, solid-state fermentation

## Abstract

**Background and Aim:**

Purple sweet potatoes (*Ipomoea batatas* var. Ayamurasaki) possess high nutritional potential due to their rich content of amino acids, minerals, and fatty acids. However, their nutritional profile can be further improved through fermentation. This study aimed to evaluate the ability of *Lactiplantibacillus plantarum* InaCC B157 to enhance the biochemical and functional composition of purple sweet potato flour.

**Materials and Methods:**

Six *L. plantarum* strains were screened for enzymatic activities. The strain with the highest amylolytic, proteolytic, and qualitative cellulolytic activity (InaCC B157) was selected for solid-state fermentation of purple sweet potato flour. Fermented *Ipomoea batatas* flour (FIB) and unfermented *Ipomoea batatas* flour (UIB) samples were analyzed for amino acid, mineral, and fatty acid content, along with vitamins A and C, dietary fiber, antioxidants, and bioactive compounds. Microstructure was examined using scanning electron microscopy (SEM). Data were statistically analyzed using a one-way analysis of variance and Duncan’s multiple range test.

**Results:**

Fermentation significantly increased the levels of essential amino acids – histidine, threonine, valine, methionine, cysteine, isoleucine, leucine, and phenylalanine (excluding lysine) – and non-essential amino acids, including glutamine, serine, glycine, and tyrosine. Mineral concentrations of zinc, calcium, potassium, magnesium, and phosphorus improved by up to 17.65%, while copper decreased. Linoleic and linolenic acids increased by 55.5% and 100%, respectively. Protein, fat, carbohydrates, fiber, and vitamins A and C also increased, while ash, anthocyanins, phenols, and steroids showed a decline. Antioxidant activity increased by 13.7%. SEM analysis revealed no substantial morphological differences between FIB and UIB.

**Conclusion:**

Fermentation of purple sweet potato flour with *L. plantarum* InaCC B157 significantly enhanced its nutritional value, particularly amino acid, mineral, and essential fatty acid profiles. These findings support the potential application of fermented purple sweet potato as a functional food and sustainable feed ingredient. Limitations include the lack of sensory evaluation and long-term stability data. Future studies should investigate sensory profiling, shelf-life extension, and optimization of fermentation parameters to further enhance the application of this functional ingredient.

## INTRODUCTION

Purple sweet potato (*Ipomoea batatas* cultivar Ayamurasaki) exhibits considerable promise as a fun-ctional food, attributable to high concentrations of amino acids, fatty acids, and minerals. Nevertheless, the application of fermentation technology is essential to further enhance its nutritional value.

Amino acids are typically derived from animal-based products such as meat, milk, and eggs, as well as plant-based sources, including legumes. These biomo-lecules are essential for both human and animal health, contributing to cytokine and antibody production, supporting the immune and cardiovascular systems, promoting muscle growth, and alleviating fatigue and dehydration. Amino acids are broadly categorized into three groups: Essential (histidine, isoleucine, leucine, lysine, methionine, phenylalanine, threonine, trypt-ophan, and valine); non-essential (glycine, alanine, argi-nine, glutamine, serine, glutamic acid, proline, cysteine, asparagine, and aspartic acid); and semi-essential, the latter of which are synthesized under normal physio-logical conditions but become essential under stress [1].

Essential amino acids play vital roles in metabolic processes, immune regulation, energy production, hemoglobin formation, and neurotransmitter synthesis, such as serotonin. As they cannot be synthesized endog-enously by humans or animals, they must be acquired through dietary intake. Conversely, non-essential amino acids are produced internally in sufficient quantities to support maintenance, growth, and development and thus do not require dietary supplementation [2].

Minerals are another critical component of the diet, serving key roles in skeletal structure, muscle and nerve function, and maintaining water homeostasis [3]. Zinc (Zn), in particular, is a vital micromineral involved in DNA replication, cellular proliferation, cognitive development, reproduction, and immune system modulation in both humans and animals [4, 5]. Macr-ominerals, such as calcium (Ca) and phosphate, are essential for neuromuscular activity, bone development, and a range of biochemical processes that rely on proper electrolyte balance [6]. Furthermore, trace elements, including iron, manganese, zinc, chromium, cobalt, copper (Cu), iodine, molybdenum, and selenium are indispensable for enzymatic activity, vitamin B12 synthesis, hemoglobin formation, and oxidative-reduct-ive metabolism.

Fatty acids act as key metabolic fuels and are primary structural components of phospholipids and glycolipids in biological membranes. They also function as precursors for hormone synthesis and intracellular signaling pathways [7]. As the main constituents of lipids, fatty acids serve both structural and energetic purposes, engaging in diverse metabolic processes [8].

Lactic acid bacteria (LAB) are microbiologically significant in food processing and preservation due to their ability to produce lactic acid, which reduces the pH and suppresses the growth of pathogenic microorganisms, thereby stabilizing food quality [9]. Among these, *Lactiplantibacillus plantarum* is widely recognized for its probiotic properties and beneficial metabolic activities.

Despite the recognized nutritional and functional potential of purple sweet potatoes (*I. batatas* var. Ayam-urasaki), existing literature reveals limited appli-cation of microbial fermentation to enhance their biochemical composition, particularly with respect to essential amino acids, bioavailable minerals, and health-promoting fatty acids. Most prior studies have focused on the antioxidant or anthocyanin profiles of sweet potato cultivars, while relatively few have investigated the use of indigenous strains of *L. plantarum* for nutrient biotransformation in solid-state fermented systems. Furthermore, data remain scarce on how specific enzy-matic activities (e.g., amylolytic, proteolytic, cellul-olytic) of locally sourced *L. plantarum* strains influence the nutritional composition of purple sweet potato flour. Although fermentation has shown promise in enhancing the digestibility and functional attributes of other starchy root crops, its targeted application to *I. batatas* var. Ayamurasaki, which uses enzymatically active LAB strains isolated from native Indonesian flora, remains largely unexplored. Moreover, the simu-ltaneous profiling of amino acids, minerals, and fatty acids following such biotransformation processes, supported by microstructural analysis, has not been comprehensively reported in previous studies. This represents a critical gap in optimizing fermentation-based interventions to valorize underutilized functional foods.

The present study aimed to evaluate the efficacy of *L. plantarum* InaCC B157, an enzymatically active strain isolated from Indonesian plant sources, in enhancing the nutritional quality of purple sweet potato (*I. batatas* var. *Ayamurasaki*) flour through solid-state fermentation. Specifically, the study sought to (i) characterize the enzymatic potential of multiple *L. plantarum* strains to identify the most suitable candidate for fermentation; (ii) quantify the changes in amino acid, mineral, and fatty acid profiles in fermented versus unfermented samples; and (iii) assess additional bioactive components, including vitamins, antioxidant capacity, and phytochemical constituents, alongside scanning electron microscopy (SEM) for morphological changes. Through this multifaceted approach, the study aims to establish the functional enhancement potential of microbial fermentation and its implications for deve-loping value-added nutritional products for human and animal applications.

## MATERIALS AND METHODS

### Ethical approval

All experiments were performed *in vitro*; therefore, ethical approval was not required for this study.

### Study period and location

This study was conducted from January to October 2024 at the Biochemistry Laboratory, Applied Micr-obiology. National Research and Innovation Agency, Bogor, Indonesia.

### Isolation and selection of *L. plantarum* InaCC strains

#### Source of isolates

The sources of *L. plantarum* isolates from various plants are listed in Table 1. *L. plantarum* was isolated from several plants, including purple passion (*Passiflora edulis*) from Lombok, West Nusa Tenggara; red sweet potatoes from Bogor, West Java; mango of Lali Jiwa (*Mangifera lalijiwa*) from Bali; *Mangifera indica* var. *Lali Jiwo* from Lombok, West Nusa Tenggara; *Annona montana* from Cibinong, West Java; and golden banana (*Musa aromatic*) from Sanggau, West Kalimantan.

**Table 1:** Enzymatic activity of *L. plantarum* strains.

Strain	Source of *L. plantarum* isolates	Enzymatic Activity		

		Amylolytic	Proteolytic	Cellulolytic
InaCC B151	*Purple Passion* (Passiflora edulis) Lombok, West Nusa Tenggara.	-	3	-
InaCC B153	Red sweet potatoes, Bogor, West Java.	-	3	-
InaCC B157	Mango of the Lali Jiwa (*Mangifera lalijiwa*), Bali.	2.8	3	+/-
InaCC B168	Mango Lali Jiwo (*Mangifera indica* var. Lali Jiwo, and Lombok West Nusa Tenggara.	-	3	-
InaCC B171	The fruit of (*Annona montana*), Cibinong, West Java.	-	3	-
InaCC B179	Golden Banana (*Musa aromatic*), Sangau, West Kalimantan.	-	3.3	-

*L. plantarum=Lactiplantibacillus plantarum*

#### Enzymatic activity assays

Amylolytic, cellulolytic, and proteolytic activities were assessed in six *L. plantarum* strains (InaCC B151, B153, B157, B168, B171, and B179) to identify the strain with the highest enzymatic activity. The strain exhibiting the highest enzymatic activity was selected for fermentation of purple sweet potato flour. To det-ect amylase, protease, and cellulase activity, cultures were inoculated into appropriate media, and the clea-rance zone was observed. The detection of amylase, protease, and cellulase activities was performed in two replications.

- Amylolytic activity: Each *L. plantarum* strain was streaked onto MRS (de Man, Rogosa, and Sharpe), agar (Oxoid, England), supplemented with 0.5% glucose and 1% starch at pH 2.5. After 48 h of incubation at 37°C, plates were flooded with 1% iodine solution for 20 min. A clear zone around colonies indicated amylase activity.
- Cellulolytic activity: Strains were streaked on MRS plates containing 1% CMC (Carboxymethyl Cellu-lose; Himedia, India). Following 48 h of incub-ation at 37°C, plates were flooded with 1% Congo red and rinsed with 0.15 N NaCl to observe clear zones.
- Proteolytic activity: Following Suciati *et al*. [10], each strain was streaked onto MRS medium supplemented with 1% skim milk and incubated at 37°C for 2 days. A clear halo around colonies indicated protease activity.

#### Preparation of purple sweet potato flour

Four-month-old purple sweet potatoes were obtained from Ciawi, Bogor, West Java. Tubers were peeled, washed thoroughly, sliced into 1-cm-thick pie-ces, drained, and oven-dried at 70°C for 7 days. The dried material was milled and sieved to a particle size of 80 mesh (177 μm), followed by Ambarsari *et al*. [11].

#### Propagation of *L. plantarum* InaCC B157

*L. plantarum* InaCC B157 was cultured on MRS agar in a Petri dish and incubated for 48 h at 37°C. A single colony was aseptically transferred into 250 mL of sterile medium (distilled water with 1% yeast extract, 2% peptone, and 1% purple sweet potato flour). This culture was incubated anaerobically at 37°C for 3 days with shaking at 100 rpm. Optical density was measured at 600 nm, and pH was recorded after 24 h.

#### Solid-state fermentation of purple sweet potato

Two hundred grams of purple sweet potato flour were placed into a sterile plastic basin (ultra-violet-sterilized and alcohol-wiped). Sterile water (250 mL) was gradually added while stirring, followed by 2.5 mL of *L. plantarum* InaCC B157 starter culture (1% v/v). The mixture was stirred, covered with gauze, and incubated at 29°C for 24 h. The resulting fermented flour was crushed with a sterile spoon and oven-dried at 40°C–50°C for 24 h to yield FIB. This sample was analyzed for amino acids, minerals, fatty acids, chemical components (vitamins A and C), phenolics, flavonoids, steroids, anthocyanins, antioxidants, and microstructure.

#### Chemical composition analysis

##### Proximate composition

Moisture, ash, protein, fat, and carbohydrate contents were determined using the Association of Official Analytical Chemists (AOAC) [12] methods. Pro-tein content was analyzed by autodestruction, fat by Soxhlet extraction, and carbohydrates, as described by Santoso *et al*. [13]. Dietary fiber was also measured. All analyses were performed in duplicate.

##### Vitamin A and C content

Vitamins A and C were quantified using high-performance liquid chromatography (HPLC), as desc-ribed by Santoso *et al*. [13]. A C-18 column was used with a methanol: water (90:10) mobile phase at a flow rate of 20 mL/min and a detection wavelength of 325 nm. Analyses were performed at room temperature (28°C) and repeated twice.

#### Amino acid profile analysis

Amino acid profiles were determined using the UltiMate 3000 HPLC System (Shimadzu AA 7000, Japan) as described by Lee *et al*. [14].

#### Mineral content determination

##### Major and trace elements

Mineral analysis followed by Santoso *et al*. [13]. Five grams of the sample was ashed at 600°C for 4 h and digested with concentrated HNO_3_. After dilution and filtration, the solution was analyzed for Ca, magnesium (Mg), potassium (K), Mn, zinc (Zn), and Cu using ato-mic absorption spectrophotometry. Measurements were performed using specific wavelengths, and concen-trations were calculated using standard calibra-tion curves.

##### Phosphorus (P) content

Phosphate levels were measured using vanadate-molybdate spectrophotometry by Ma *et al*. [15]. The ashes were treated with nitric acid, filtered, and then reacted with a vanadate-molybdate reagent. Absorbance was measured at 410 nm. Analyses were conducted in duplicate.

#### Fatty acid composition analysis

Fatty acids were analyzed using gas chromat-ography as per AOAC [12]. The analysis was performed in duplicate.

#### Determination of monomeric anthocyanins

Anthocyanins were extracted according to Rahmawati *et al*. [16] using 5% HCl in methanol. Extracts were concentrated through rotary evaporation and analyzed using the pH differential method as described by Liu *et al*. [17]. Concentrations were expressed in mg cyanidin-3-glucoside equivalent per gram of dry matter. Each analysis was conducted twice.

#### Analysis of functional components

##### Extraction procedure

Ten grams of UIB and FIB were extracted with 50 mL of methanol at 120 rpm for 3 h at 29°C. Filtrates were collected through Whatman filter paper No. 42 (Merck, England) using a Buchner funnel and evaporated at 40°C (50 mm Hg). Extracts were either analyzed immediately or stored at 4°C.

##### Antioxidant activity

Antioxidant activity was assessed using the DPPH radical scavenging method, as described by Basrin *et al*. [18].

##### Total phenolics

Total phenolics were measured using the Folin–Ciocalteu reagent method described by Im *et al*. [19].

##### Flavonoid content

Flavonoid levels were determined by Tasminto *et al*. [20] with minor modifications. Extracts were reacted with AlCl_3_ and acetic acid, incubated, and absorbance measured at 410 nm. Results were expr-essed as quercetin equivalents (mg QE/kg extract).

Flavonoids (mg QE/g extract) = (C × V × df)/g

Where:

C = flavonoid concentration (x value),

V = volume of sample extract (mL),

df = dilution factor,

g = sample weight (g).

##### Steroid content

Steroids were quantified following Hardiningsih and Novik [21] using a chloroform-acetic acid-sulfuric acid reaction. After incubation, absorbance was measured at 366 nm using a cholesterol standard.

#### SEM

The morphological characteristics of UIB and FIB were analyzed using SEM JSM-5000, as described by Yong *et al*. [22]. Micrographs were captured at 1000× magnification with an accelerating voltage of 20 kV and an image length of 132 μm.

#### Statistical analysis

All data were subjected to one-way analysis of variance using SAS software (version 9.4; SAS Institute, NC, USA). Duncan’s multiple range test was applied to determine significant differences among treatments at p < 0.05 due to its high sensitivity when comparing multiple means.

## RESULTS

### Selection of enzymatically active *L. plantarum* strains

**Figure 1:**
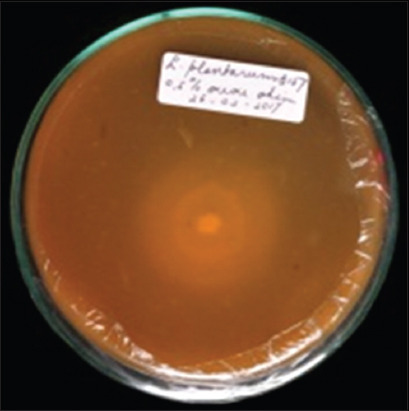
Proteolytic activity of *Lactiplantibacillus plant-arum* InaCC B157.

**Figure 2:**
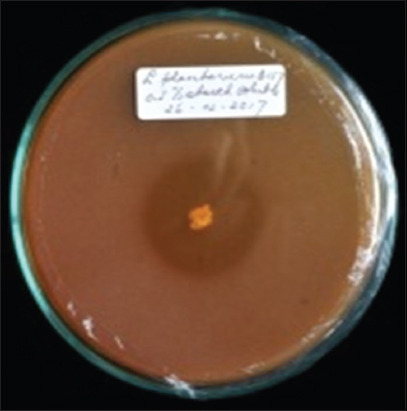
Amylolytic activity of the *Lactiplantibacillus plantarum* InaCC B157.

**Figure 3:**
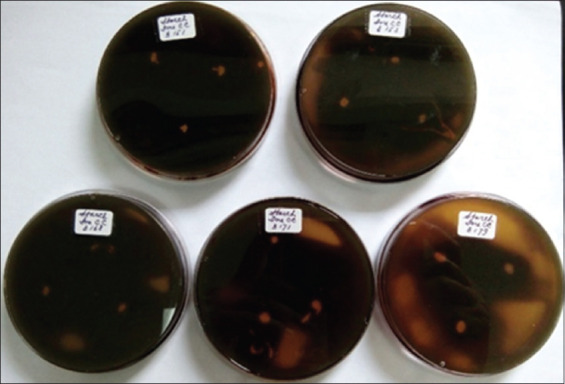
Amylolytic activity of the *Lactiplantibacillus plantarum* InaCC B151, B153, B168, B17, and B179.

**Figure 4:**
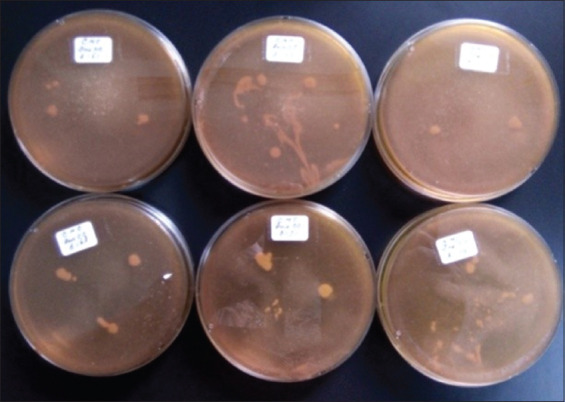
Cellulolytic activity of *Lactiplantibacillus plantarum* InaCC B151, B153, B157, B168, B171, and B179.

*L. plantarum* strains were isolated from plants collected across six regions in Indonesia, including *P. edulis* from Lombok, red sweet potato from Bogor, *M. lalijiwa* from Bali, *M. indica* var. *Lali Jiwo* from Lombok, *A. montana* from Cibinong, and *M. aromatic* from West Kalimantan (Table 1). Among the tested strains, *L. plantarum* InaCC B157, originating from *M. lalijiwa*, demonstrated the highest enzymatic activity, with clear zones of 3 mm and 2.8 mm for proteolytic and amylolytic activities, respectively (Table 1, Figures 1 and 2). Cellu-lolytic activity in this strain was qualitatively detected. Other strains (InaCC B151, B153, B168, B171, and B179) exhibited strong proteolytic activity with clear zones ranging from 3 to 3.8 mm but lacked amylolytic and cellulolytic activity (Figures 3 and 4). The observed enzymatic activities were indicated by clear zones surrounding bacterial colonies. Based on its superior enzyme profile, InaCC B157 was selected as the starter culture for the fermentation of purple sweet potato flour.

### Amino acid profile enhancement

**Figure 5:**
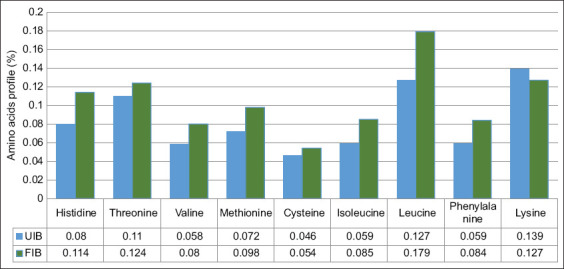
Changes in the essential amino acid profiles of fermented and unfermented purple sweet potatoes. UIB=Unfermented *Ipomoea batatas* flour, FIB=Fermented *Ipomoea batatas* flour.

**Figure 6:**
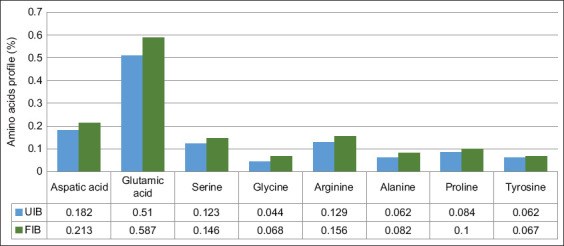
Changes in nonessential amino acid profiles in fermented versus unfermented purple sweet potatoes. UIB=Unfermented *Ipomoea batatas* flour, FIB=Fermented *Ipomoea batatas* flour.

Essential amino acids, such as histidine, isoleucine, leucine, lysine, methionine, phenylalanine, threonine, tryptophan, and valine, cannot be synthesized endog-enously and must be acquired through the diet. Conve-rsely, non-essential amino acids, including arginine, tyrosine, cysteine, glycine, proline, serine, and ornithine, are synthesized within the body. In this study, nine essential amino acids were detected in purple sweet potato flour: Histidine, threonine, valine, cysteine, methionine, isoleucine, leucine, phenylalanine, and lysine. Fermentation with *L. plantarum* InaCC B157 significantly increased the concentrations of nearly all essential amino acids in the FIB samples (Figure 5). This enhancement is attributed to proteolytic enzymes secreted by *L. plantarum*, which hydrolyze proteins into free amino acids. Among the non-essential amino acids, glutamic acid exhibited the highest concentration, followed by aspartic acid, serine, arginine, proline, alanine, tyrosine, and glycine (Figure 6).

### Mineral content modification

Fermentation significantly (p < 0.05) increased the levels of Zn, Ca, K, Mg, and P in purple sweet potato flour, while Cu content decreased (Table 2). The highest improvement was recorded in P (17.65%), followed by Ca (15.8%), Zn (12.1%), Mg (4.67%), and K (0.12%). Cu content declined by 9.3%.

**Table 2:** Changes in mineral profiles between fermented and unfermented purple sweet potato flour.

Types of minerals (mg/100 g)	UIB	FIB	Improvement (%)
Zinc (Zn)	0.58	0.65	12.1
Calcium (Ca)	38.99	45.16	15.8
Potassium (K)	339.65	340.07	0.12
Magnesium (Mg)	26.12	27.34	4.67
Phosphor (P)	61.32	72.14	17.65
Cuprum (Cu)	0.43	0.39	−9.3

UIB=Unfermented *Ipomoea batatas* flour, FIB=Fermented *Ipomoea batatas* flour

### Fatty acid composition shifts

**Figure 7:**
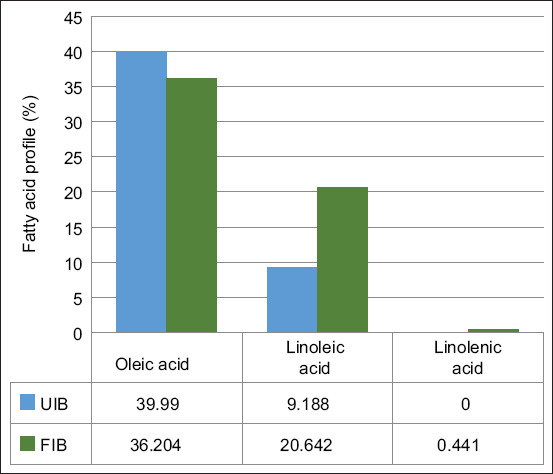
Changes in the essential fatty acid profiles of fermented and unfermented purple sweet potato. UIB=Unfermented *Ipomoea batatas* flour, FIB=Fermented *Ipomoea batatas* flour.

**Figure 8:**
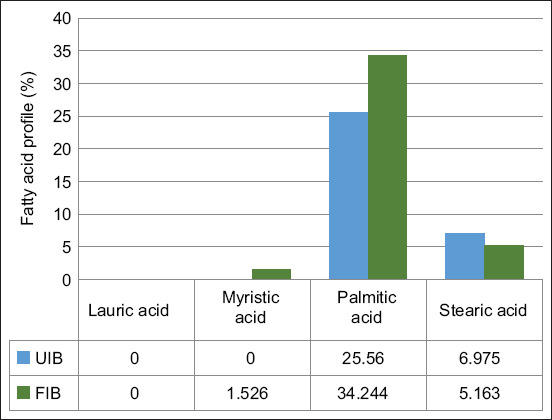
Changes in non-essential fatty acid profiles in fermented versus unfermented purple sweet potatoes. UIB=Unfermented *Ipomoea batatas* flour, FIB=Fermented *Ipomoea batatas* flour).

Fermentation with *L. plantarum* InaCC B157 led to significant increases (p < 0.05) in the essential fatty acids linoleic acid and linolenic acid, by 55.5% and 100%, respectively. Conversely, oleic acid content decreased by 10.5% (Figure 7). Among the non-essential fatty acids, myristic acid and palmitic acid increased significantly by 100% and 25.4%, respectively. Stearic acid decreased by 35.1%, while lauric acid levels remained unchanged (Figure 8).

### Changes in nutritional composition

As summarized in Table 3, fermentation enhanced the macronutrient profile of purple sweet potato flour. In FIB, protein, fat, carbohydrate, and fiber content increased by approximately 3.8%, 8.7%, 2.8%, and 17.3%, respectively. In addition, vitamin A and C levels increased, while ash content showed a slight decrease after fermentation.

**Table 3:** Nutrient compositions of fermented and unfermented purple sweet potato flour.

The type of analysis	UIB	FIB	Improvement (%)
Moisture (%)	92.7	95.32	2.83
Protein (%)	2.40	2.49	3.8
Fat (%)	1.03	1.12	8.7
Carbohydrate (%)	88.04	90.53	2.8
Fiber (%)	4.79	5.62	17.3
Vitamin A (SI)	7.97	8.38	5.1
Vitamin C (mg/100 g)	32.29	35.69	10.5
Ash (%)	1.22	1.19	−2.7
Anthocyanin (mg/100 g)	155.17	129.83	−16.3

UIB=Unfermented *Ipomoea batatas* flour, FIB=Fermented *Ipomoea batatas* flour

### Phytochemical and antioxidant activity

Fermentation influenced the production of various bioactive compounds in purple sweet potatoes (Table 4). While total phenolic and steroid contents decreased in FIB, antioxidant activity increased, with inhibition levels rising from 38.73% (UIB) to 44.03% (FIB). Alkaloid content also increased, although specific values were not provided.

**Table 4:** Changes in phenol, flavonoids, steroids, and antioxidant inhibition in fermented versus unfermented purple sweet potato flour.

Phytochemical content	UIB	FIB	p-value
Phenolic (mg GAE/g)	0.36^a^	0.28^b^	0.0465
Flavonoids (mg QE/g)	0.13	0.16	0.4685
Steroid (mg/g)	0.04	0.03	0.1161
Antioxidant (% Inhibition)	38.73^b^	44.03^a^	0.0230

UIB=Unfermented *Ipomoea batatas* flour, FIB=Fermented *Ipomoea batatas* flour Different superscript (a–g) within the same row indicates significantly different (p < 0.05).

### Microstructural characteristics

**Figure 9:**
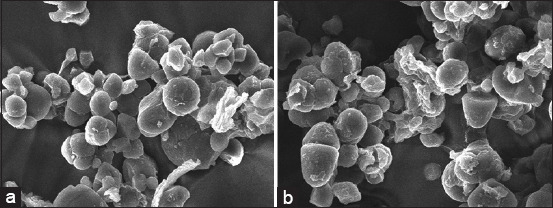
(a) SEM analysis of unfermented *Ipomoea batatas* flour and (b) SEM analysis of fermented *Ipomoea batatas* flour. SEM=Scanning electron microscopy.

SEM images of UIB and FIB (Figures 9a and 9b) revealed no distinct morphological differences. Starch granules in both samples exhibited variability in size, from small to large, and diverse shapes, including oval, round, and irregular forms.

## DISCUSSION

### Enzymatic classification and fermentative role of *L. plantarum* InaCC B157

According to established enzymatic classification, activity levels are defined as low (0–1 mm), medium (1–2 mm), and high (>2 mm) [23]. *L. plantarum* InaCC B157 was categorized as exhibiting high enzymatic activity. Consequently, this strain was selected as the inoculum for the fermentation of purple sweet pota-toes. During the fermentation process, LAB, such as *L. plantarum*, metabolize carbohydrates into lactic acid and produce beneficial metabolites, including short-chain fatty acids, amines, bacteriocins, vitamins, and exopolysaccharides [24].

The enzymatic profile of InaCC B157 demonstrated concurrent amylolytic, proteolytic, and cellulolytic capa-bilities, categorizing it as a multifunctional hydrolytic strain. In contrast, strains InaCC B151, B153, B168, B171, and B179 exhibited only proteolytic activity, indicating that the media formulation may have been suboptimal for inducing amylase and cellulase in these strains. Optimal growth of *L. plantarum* InaCC B157 occurred on the 3^rd^ day of incubation using media supplemented with 1% purple sweet potato flour (Table 5), with a final stable pH of 4.1, thus defining the ideal incubation parameters for starter culture preparation.

**Table 5:** Growth of *L. plantarum* InaCC B157 in medium supplemented with 1% purple sweet potato flour.

Parameter	*L. plantarum* InaCC157 (day)		

	1	2	3
Number of cells (cfu/mL)	4.9×10^7^	5.2×10^8^	8.1×10^8^
pH	4.35	4.52	4.11

*L. plantarum=Lactiplantibacillus plantarum*

### Nutritional improvements through solid-state fermentation

#### Macronutrient and vitamin enhancement

The nutrient profiles of unfermented UIB and fermented FIB purple sweet potato flour are detailed in Table 3. Protein enhancement in FIB was attributed to microbial biomass accumulation, as *L. plantarum* InaCC B157 utilized water-soluble carbohydrates as fermentation substrates. This fermentation process also led to increased levels of fat, carbohydrates, and dietary fiber, while the ash content declined slightly. The moisture content remained high in both UIB and FIB, consistent with the intrinsic high water content of purple sweet potatoes, which is attributed to their expansive intercellular spaces [25, 26]. Vitamin A and C content in FIB increased by 5.1% and 10.5%, respectively, supporting its value as a functional food for humans and livestock.

### Amino acid modulation

Fermentation with InaCC B157 significantly increased the levels of most essential amino acids in FIB, including histidine, threonine, valine, methionine, cysteine, isoleucine, leucine, and phenylalanine. Lysine, however, decreased – likely consumed by *L. plantarum* for growth and energy metabolism [12]. Among non-essential amino acids, aspartic acid, glutamine, serine, glycine, arginine, alanine, proline, and tyrosine increased.

The essential amino acid content of FIB rose to 0.95%, a 26% increase compared to UIB (0.75%). Similarly, non-essential amino acids increased from 1.196% in UIB to 1.419% in FIB – approximately a 19% rise. This enrichment is attributed to the enzy-matic hydrolysis of proteins and possible microbial synthesis [27]. The increase in amino acids, especially essential ones, aligns with feed formulation standards for poultry, where enhanced protein quality directly improves growth and production performance [28–30].

Furthermore, microbial preferences in amino acid metabolism explain the lower increase in glutamate and aspartate, which are often utilized as sources of carbon and nitrogen. *L. plantarum* is known to consume valine, lysine, cysteine, threonine, and arginine as preferred growth substrates [31, 32].

### Mineral composition dynamics

The P content of FIB increased post-fermentation, supporting its role in bone, DNA, RNA synthesis, enzyme regulation, and energy storage [33, 34]. Ca levels also rose, reinforcing its biological necessity for skeletal development, cardiac function, and cellular signaling, with 99% stored in bones and 1% distributed in blood and tissues [34].

Although Cu levels in FIB decreased, it remains a functional food candidate. Cu plays a key role in enzymatic activity, connective tissue synthesis, immune modulation, and energy metabolism [35–38]. The redu-ction in Cu is likely due to bacterial utilization during metabolic processes.

### Fatty acid profile alterations

Lipase activity from *L. plantarum* InaCC B157 contributed to increased levels of linoleic acid, linolenic acid, myristic acid, and palmitic acid, generated through microbial lipolysis [39]. Notably, linoleic acid was not converted into stearic acid, contrary to findings with other *Lactobacillus* species [40].

*L. plantarum* also modulates lipid metabolism through the production of SCFAs and the suppression of endogenous fatty acid synthesis genes [41, 42]. However, the levels of oleic acid and stearic acid decreased, highlighting specific lipid transformation pathways active in this fermentation model.

### Phytochemical and antioxidant shifts

The total phenolic and steroid content in FIB declined. This contrasts with other *L. plantarum* ferme-ntation studies (e.g., avocado leaves), indicating that substrate type and bacterial strain specificity affect phenolic outcomes [43]. Conversely, antioxidant activity and alkaloid content increased, with FIB showing a 13.7% enhancement in antioxidant capacity (Table 4). This is consistent with prior studies reporting elevated antioxidant properties in vegetable-based beverages fermented with *L. plantarum* [44].

Fermentation retains or improves anthocyanin stability in purple sweet potatoes, which is critical for antioxidant activity, although the effects depend on variables such as sugar content, strain, pH, and dura-tion [45–50].

### Functional food and feed applications

FIB demonstrates potential as a dual-use functional food and feed additive. Fermentation increases digestibility and bioavailability while adding probiotics and bioactive compounds. FIB can be formulated into yogurt [51], snacks [52], or poultry feed to enhance growth performance [53], promoting sustainable livest-ock production.

### Morphological analysis by SEM

SEM revealed no significant differences in starch granule morphology between UIB and FIB (Figures 9a and b). Granules maintained their original size and shape (oval, round, irregular), indicating mini-mal enzymatic hydrolysis. This suggests that enzymatic activity, although present, was insufficient to alter the physical matrix of starch granules during the ferme-ntation period [54].

## CONCLUSION

This study demonstrated that *L. plantarum* InaCC B157, isolated from *M. lalijiwa* fruit, exhibits high amylolytic, proteolytic, and moderate cellulolytic activities, qualifying it as an effective fermentative starter for enhancing the nutritional profile of purple sweet potato (I. cv. Ayamurasaki). The fermentation process significantly increased the concentrations of essential amino acids (except lysine); non-essential amino acids, critical minerals such as P, Ca, Zn, Mg, and K; essential fatty acids including linoleic and linolenic acid; and antioxidant activity, while reducing undesirable elements, such as Cu and residual moisture. Moreover, the levels of protein, fat, carbohydrate, fiber, and vitamins A and C also improved in the fermented product.

The application of *L. plantarum* InaCC B157 in solid-state fermentation presents a promising biotechnological strategy for valorizing purple sweet potato as a cost-effective, nutrient-enriched, and funct-ional food or livestock feed ingredient. Its improved nutritional properties support potential use in the development of high-protein poultry feed formulations and functional food products such as probiotic yogurts or health-oriented snacks, particularly in resource-limited settings.

Among the major strengths of this study are the comprehensive biochemical analyses encompassing amino acids, fatty acids, minerals, vitamins, and antioxidant profiles; the use of a native bacterial strain with demonstrable enzymatic activity; and the application of SEM to assess structural effects on starch granules. Nonetheless, some limitations were observed. The study did not evaluate the sensory characteristics, palatability, or storage stability of the fermented prod-uct. The lysine content decreased, which may impact the amino acid balance in feed applications. In addition, morphological changes in the starch granules were not observed, despite the presence of enzymatic activity, suggesting that starch degradation was limited under the given conditions.

Future investigations should aim to optimize fermentation parameters such as duration, temperature, and substrate ratio to enhance lysine preservation and promote more extensive modification of starch structures. *In vivo* trials are also warranted to evaluate the digestibility and physiological benefits of fermented purple sweet potato in livestock and human populations. Moreover, co-fermentation with additional probiotic strains could be explored to improve synergistic metabolic outputs and overall product functionality.

In conclusion, the fermentation of purple sweet potatoes using *L. plantarum* InaCC B157 is an effective method for improving nutritional and functional att-ributes, supporting their dual use as both dietary supplements and sustainable animal feed components. This biotransformation strategy enhances the value of indigenous crops and contributes to the advancement of microbial biotechnology in food and feed innovation.

## AUTHORS’ CONTRIBUTIONS

YSS: Designed the study and collected data. YSS and TP: Interpreted the data and wrote the original manuscript. YSS, TP, and NN: Validated and investigated the data. YSS, TP, NN, SS, TY, ET, TRS, NS, DDS, and DNS: Statistical analysis and edited and revised the manuscript. All authors have read and approved the final manuscript.
